# Primary Autologous Stem Cell Transplantation for Unilateral Primary Central Nervous System Lymphoma–Ophthalmic Variant (Primary Vitreoretinal Lymphoma)

**DOI:** 10.1177/24741264231174094

**Published:** 2023-06-05

**Authors:** Brian Lee, Sven de Vos, Colin A. McCannel

**Affiliations:** 1 Department of Ophthalmology, Stein Eye and Doheny Eye Institutes, University of California, Los Angeles, CA, USA; 2 Division of Hematology and Oncology, David Geffen School of Medicine at the University of California Los Angeles, Los Angeles, CA, USA; 3 Department of Ophthalmology, Stein Eye Institute, University of California, Los Angeles, CA, USA

**Keywords:** primary vitreoretinal lymphoma, primary central nervous system lymphoma, autologous stem cell transplantation, primary central nervous system lymphoma–ophthalmic variant

## Abstract

**Purpose:** To describe the long-term outcomes of 2 cases of primary autologous stem cell transplantation (ASCT) for the treatment of primary central nervous system lymphoma–ophthalmic variant (PCNSL-O) or primary vitreoretinal lymphoma (PVRL). **Methods:** Two cases and their findings were analyzed. A review of the histopathology, systemic treatment, and multimodal ocular imaging was performed. **Results:** A 52-year-old woman and 56-year-old woman were referred for vitritis and retinal lesions suspicious for PCNSL-O. The initial vitreous biopsies were inconclusive. Both patients had subsequent chorioretinal biopsies that confirmed the diagnosis of diffuse large B-cell lymphoma. No systemic or central nervous system involvement was found on systemic workup. Both patients received intravitreal and systemic chemotherapy followed by ASCT, and both remained in complete remission 7 and 8 years later. **Conclusions:** These cases show the long-term survival of patients diagnosed with PVRL when primary ASCT, the primary treatment for PCNSL, is performed.

## Introduction

Primary vitreoretinal lymphoma (PVRL) is a fatal malignancy more appropriately described as primary central nervous system lymphoma–ophthalmic variant (PCNSL-O). Although previously known as primary intraocular lymphoma (PIOL) or ocular reticulum cell sarcoma, the disease is not limited to the eye. Local intravitreal methotrexate treatment invariably results in 75% to 87% of patients showing CNS involvement within 4 years.^1,2^

The PRECIS randomized control trial showed 4-year overall survival of 85% in PCNSL patients receiving MATRix induction and autologous stem cell transplantation (ASCT).^3,4^ Both the PRECIS and IELSG32 trials found that using multiple agents for induction chemotherapy followed by ASCT, which restores hematopoiesis using filtered autologous stem cells after intensive consolidation chemotherapy, achieves better remission in PCNSL.^3,4^ However, less than 40% of oncologists and ophthalmologists use systemic treatment as a first-line treatment for PCNSL-O.^5^ This is likely because older retrospective studies of PCNSL-O that was diagnosed before 2005 found no survival benefit for systemic treatment over local treatment; at that time, the 4-year survival was below 60%.^1^ Although the treatment of PCNSL has advanced, patients with PCNSL-O have been excluded from recent trials.^3,4^

PCNSL-O historically presented bilaterally in 64% to 83% of cases; however, recent reports indicate bilateral presentation in only 25% of cases.^6,7^ The increasing incidence of unilateral PCNSL-O presentation could be related to earlier diagnosis. However, the overall survival for unilateral PCNSL-O is 24 months shorter than for bilateral PCNSL-O, and the recommendation for limited local treatment of unilateral PCNSL-O may be related to the worse systemic outcomes in unilateral disease.^8,9^

Although ASCT has been studied only as a salvage therapy in PCNSL-O, the similar outcomes of PCNSL-O and PCNSL show it is likely to be beneficial as a primary treatment for PCNSL-O.^10^ PCNSL-O should be treated as PCNSL and should not be excluded from PCNSL trials.^3,4^

We report 2 cases of PCNSL-O treated with primary ASCT.

## Case Reports

### Case 1

**Figure 1.**
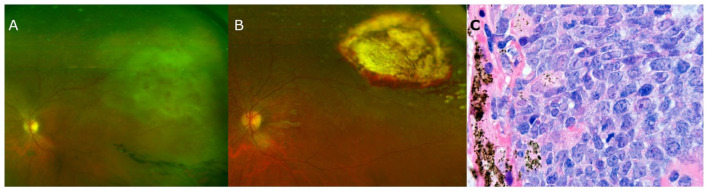
(A) Ultra-widefield fundus photograph shows vitritis and a yellow–white retinal lesion. (B) Ultra-widefield fundus photograph shows peripheral chorioretinal scarring 6 months after the chorioretinal biopsy. (C) Histopathology shows diffuse infiltration of large atypical lymphoid cells consistent with diffuse large B-cell lymphoma.

A 52-year-old woman presented reporting floaters in the left eye. She had dense vitritis and a yellow–white retinal lesion, unresponsive to antiviral treatment (Figure 1A). A vitreous biopsy was suggestive of, but not diagnostic for, PCNSL-O. A subsequent chorioretinal biopsy (Figure 1B) showed diffuse large B-cell lymphoma expressing CD20, multiple myeloma 1, B-cell lymphoma 2, B-cell lymphoma 6 (BCL-6), and *MYC* proto-oncogene and was negative for CD10 and CD3 (Figure 1C). There was no other systemic involvement on contrast-enhanced magnetic resonance imaging (MRI) and lumbar puncture.

The patient received 4 cycles of high-dose methotrexate, rituximab, and leucovorin rescue, followed by 2 cycles of rituximab, cytarabine, and thiopeta, followed by busulfan, cyclophosphamide, and thiopeta. She also received 2 doses of intravitreal methotrexate. ASCT was completed 4 months after diagnosis. The patient received annual neuroimaging, and she remained in complete remission 8 years later with a visual acuity (VA) of 20/30.

### Case 2

A 56-year-old woman presented for photopsias and shadows in the left eye. She had grade 1 vitritis and a 3-disc-diameter multifocal yellow–white retinal lesion in the periphery. The vitreous biopsy was nondiagnostic. A subsequent chorioretinal biopsy was positive for CD10, CD20, BCL-6, and *MYC* proto-oncogene. There was no other systemic involvement on contrast-enhanced MRI and lumbar puncture.

The patient received 2 cycles of high-dose methotrexate, rituximab, and leucovorin rescue and then 2 reduced cycles with 3 mg/m^2^ methotrexate, followed by 2 cycles of rituximab, cytarabine, and thiopeta, followed by busulfan, cyclophosphamide, and thiopeta. She also received 4 doses of intravitreal methotrexate. ASCT was completed 4 months after diagnosis. The patient received annual neuroimaging, and she remained in complete remission 7 years later with a VA of 20/400 in the affected eye.

## Conclusions

PCNSL-O is PCNSL that is first diagnosed in the eye. This terminology better describes the disease as PIOL and PVRL, both of which imply that the disease originates in the eye.^8^ This shift in terminology suggests that PCNSL-O should be treated as PCNSL.

The increasing frequency at which PCNSL-O is diagnosed in 1 eye rather than both eyes is likely a result of earlier diagnosis stemming from increased awareness and better diagnostics. Progression to bilateral or CNS involvement is the natural course of PCNSL and occurs in 75% to 87% of patients by 4 years.^6,7^ PCNSL or bilateral PCNSL-O is often found during workup; however, unilateral PCNSL-O makes up a small but increasing fraction of all PCNSL and PCNSL-O cases.^1,9,11^ Although one would expect unilateral disease to be less severe, a recent French nationwide database showed that unilateral PCNSL-O had a worse prognosis (51-month median survival) than bilateral disease (75-month median survival).^9^ The worse outcome for unilateral disease than for bilateral disease may represent undertreatment. Thus, unilateral PCNSL-O is a sign of PCNSL and might require a more aggressive treatment approach, similar to that for bilateral PCSNL-O and PCNSL.

Systemic treatment of PCNSL has improved in the past 20 years, and these advances should be used in the treatment of PCNSL-O to prevent relapse.^12^ Previous retrospective studies of PCNSL-O patients diagnosed between 1977 and 2005 found no survival benefit for patients treated with systemic chemotherapy or radiation vs local chemotherapy.^1^

Patients often show a good initial response to local treatment; thus, oncologists and ophthalmologists were hesitant to recommend systemic treatment for unilateral PCNSL-O and instead used more conservative treatment with local therapy, such as intravitreal methotrexate or rituximab, to avoid the toxicity of systemic chemotherapy.^5,6^ In a recent survey, less than 40% of oncologists and ophthalmologists used systemic treatment as a first-line treatment for PCNSL-O.^5^ As the treatment of PCNSL advances, this could lead to worse outcomes for PCNSL-O than for PCNSL.

The recognition that PCNSL-O almost inevitably progresses to PCNSL has led to renewed interest in using systemic therapy for primary treatment of PCNSL-O. Although systemic methotrexate may reduce the rate of CNS involvement, there is minimal overall survival benefit compared with local therapy.^1,2,9^ The rate of relapse with patients treated with methotrexate monotherapy is high; thus, systemic or local methotrexate is insufficient.^8,9^

The general consensus for the past 5 years is that PCNSL patients younger than 70 years should be treated with consolidation therapy.^13^ There is debate about whether whole brain radiotherapy (WBRT), ASCT, or non-myeloalabative chemotherapy is the most effective consolidation therapy after high-dose methotrexate induction therapy. However, more aggressive induction therapy and more aggressive consolidation therapy have generally been related to decreased relapse and increased overall survival in PCNSL.^3^ Unfortunately, primary ASCT for PCNSL-O has not been studied, even though it has been extensively examined for PCNSL.

Despite the similarly high relapse rates and poor outcomes of PCNSL-O, PCNSL-O has been excluded from PCNSL clinical trials. The IELSG32 and PRECIS trials showed that WBRT or ASCT has a 4-year overall survival of 64% to 85%.^3,4^ Both trials specifically excluded patients with PCNSL-O.^3,4^ Even though PCNSL-O is PCNSL first diagnosed in the eye and treatment of PCNSL has shifted to using ASCT as the first-line treatment because of the high relapse rates, ASCT has been studied only as a salvage therapy for PCNSL-O.^10^

ASCT has previously been shown to be beneficial in PCNSL-O only as a salvage therapy. In a prospective phase II study of ASCT as salvage therapy for 43 patients who had failed primary treatment, Soussain et al^10^ included 5 patients with isolated intraocular lymphomas and 1 with only intraocular and cerebrospinal fluid involvement. There was no statistically significant difference in overall or progression-free survival between patients with isolated PCNSL-O and those with PCNSL. However, there was significant improvement in survival when ASCT was used as a salvage therapy. To establish optimal treatment regimens for PCNSL-O and potentially achieve similar survival improvements as seen in PCNSL, future trials of primary treatment of PCNSL may have to include patients with PCNLS-O.

Unilateral PCNSL-O is PCNSL first diagnosed in the eye. Although there is a delay of 1 to 72 months between eye diagnosis and CNS involvement, progression is almost inevitable and PCNSL-O should be treated aggressively like PCNSL.^14^ Retrospective studies have shown methotrexate is insufficient to control PCNSL-O.^1^ In multiple trials, ASCT has been found to be much more effective in treating PCNSL.^3,4^ Although PCNSL-O is less common than PCNSL, it will increase with growing lymphoma rates and earlier PCNSL-O diagnosis.^12^ Unfortunately, unilateral PCNSL-O has a worse survival rate than bilateral PCNSL-O.^9^ This difference may be the result of undertreatment because only local treatment is typically recommended for unilateral PCNSL-O.^15^ Thus, aggressive treatments such as ASCT should be offered to unilateral PCNSL-O patients to prevent relapse and achieve similar outcomes to those of bilateral PCNSL-O and PCNSL.^11^ Because PCNSL-O is PCNSL presenting in the eye, we recommend inclusion of unilateral PCNSL-O in clinical trials of the primary treatment of PCNSL.
